# Molecular Weight-Dependent Functional and Antioxidant Properties of Glutamine-Rich Corn Protein Hydrolysate: Insights into the Predicted Structural Characteristics of Identified Peptides

**DOI:** 10.3390/foods15183283

**Published:** 2026-09-17

**Authors:** Yan Jing, Yating He, Zedan Liu, Nanxin Hong, Xiaolan Liu, Jinyu Wang

**Affiliations:** 1 Heilongjiang Provincial Key Laboratory of Corn Deep Processing Theory and Technology, College of Food and Bioengineering, Qiqihar University, Qiqihar 161006, China; foodjingyan@126.com (Y.J.);; 2 Engineering Research Center of Plant Food Processing Technology, Ministry of Education, Qiqihar 161006, China

**Keywords:** corn glutamine peptide, ultrafiltration grading, structural characterization, functional property, antioxidant activity

## Abstract

In this study, corn protein hydrolysates (CPT) were prepared via enzymatic hydrolysis using Alcalase and Protamex, and then fractionated by ultrafiltration into three molecular weight components: >5 kDa (CPT1), 3–5 kDa (CPT2), and <3 kDa (CPT3). The physicochemical, functional and in vitro antioxidant properties of each component were systematically compared. Subsequently, glutamine peptides were isolated and identified from CPT3 using LC-MS/MS technology, and the structural characteristics of the identified peptides were further analyzed. The results showed that the ultrafiltration fractionation effectively enriched peptides with lower molecular weights from the enzymatically hydrolyzed products, which may potentially enhance their bioavailability. In addition, CPT3 exhibited superior foaming ability, emulsifying ability, dispersibility, and zeta potential, along with lower particle size and viscosity. Moreover, CPT3 exhibited strong antioxidant activity, with IC_50_ values for ABTS radical scavenging and hydroxyl radical scavenging of 0.042 ± 0.003 mg/mL and 0.807 ± 0.019 mg/mL, respectively. Finally, 205 glutamine peptides were further identified from CPT3, and their structural characterization revealed that the identified peptides were rich in hydrophobic amino acids and had a high affinity for membrane proteins, which could serve as potential ligands for regulating intestinal barrier dysfunction. In summary, CPT3 possesses excellent functional properties and antioxidant activity, indicating its potential for development and application in functional and health foods.

## 1. Introduction

Glutamine (Gln) is a conditionally essential amino acid in the human body, yet its application is limited by low stability and solubility [1]. To overcome this limitation, Glutamine peptides have gained attention, as they exhibit various physiological functions, including reducing intestinal damage and exerting antioxidant effects [2,3]. For instance, alanine-glutamine has been shown to alleviate intestinal damage induced by soy saponins in zebrafish, as well as to mitigate liver ischemia–reperfusion injury and attenuate fatty hepatitis and fibrosis in mice by regulating oxidative stress and inflammatory responses [4,5,6]. Therefore, the development of glutamine-rich bioactive peptides represents a promising strategy to address the application limitations of free glutamine.

Corn gluten meal, a by-product of the corn deep-processing industry, is rich in glutamine [7]. Corn protein hydrolysates derived from this material have attracted increasing attention in the food and pharmaceutical sectors due to their excellent biological activities, aligning well with the growing consumer demand for natural and healthy products [8,9]. Previous studies have reported the antioxidant and intestinal barrier protective potential of corn protein hydrolysate [10,11]. However, there is currently a lack of systematic investigations into the comprehensive properties of corn protein hydrolysates rich in glutamine peptides, as well as their fractions with varying molecular weights. Given that peptides with different molecular weight ranges often exhibit distinct properties depending on their chain length, hydrophobicity, and amino acid sequences [12], such fractionation studies are essential for understanding structure–function relationships. Tang et al. reported that the <3 kDa ultrafiltration fraction of zein hydrolysates exhibited the highest antioxidant activities in all relevant assays [13]. Similarly, in glutamine-rich corn protein hydrolysates, most glutamine-containing peptides were enriched in fractions with molecular weights below 5 kDa, and the <3 kDa fraction demonstrated superior radical scavenging capacity [14]. Therefore, ultrafiltration membranes with a molecular weight cut-off of 5 kDa and 3 kDa were selected for this study.

Our previous studies have found that corn protein hydrolysates prepared by hydrolysis with Alcalase and Protamex has the properties of alleviating inflammatory responses, protecting the intestinal barrier, and antioxidation [11,15]. Nevertheless, the specific peptide sequences responsible for these biological activities remain unclear, and their stability during gastrointestinal digestion has not been systematically investigated.

Therefore, the objective of this study was to clarify the relationship between molecular weight, structural characteristics, functional properties, and antioxidant activity of corn protein hydrolysates, and to identify glutamine-containing peptides with potential intestinal barrier-protective effects that are resistant to gastrointestinal digestion. Corn protein hydrolysates were prepared by dual-enzymatic hydrolysis and fractionated by ultrafiltration into different molecular weight fractions. Each fraction was systematically evaluated for its physicochemical properties, functional characteristics, and in vitro antioxidant activity. Based on the correlation between antioxidant capacity and intestinal barrier protection, fractions with potential intestinal barrier-protective effects were preliminarily screened [16]. The selected fractions were then subjected to in vitro simulated gastrointestinal digestion, and the peptide profiles before and after digestion were identified by LC-MS/MS to screen for glutamine-containing peptides resistant to gastrointestinal digestion. Finally, the physicochemical properties and structural characteristics of these digestion-resistant peptides were characterized in silico to prioritize promising candidates for future targeted validation.

## 2. Materials and Methods

### 2.1. Materials

Corn gluten meal (CGM) was purchased from Longfeng Corn Development Co., Ltd. in Heilongjiang Province (Suihua, China), with a protein content of 60–70%. Alcalase (31,000 U/mL) and Protamex (30,000 U/g) were purchased from Novo Nordisk (Bagvaard, Denmark). All the other reagents and chemicals were of analytical grade.

### 2.2. Preparation of CPT

CGM was pretreated via extrusion expansion followed by starch removal, using the method described in the previous study [15]. Subsequently, the pretreated CGM was sequentially hydrolyzed with Alcalase (64 °C, 2.5 h, 1300 U/g, pH 8.0) and Protamex (50 °C, 2.5 h, 200 U/g, pH 7.0) following the optimized protocol from our previous study [15]. After hydrolysis, the sample was centrifuged at 12,100× *g* (8000 rpm) for 15 min, and the supernatant was collected for subsequent analysis.

### 2.3. Ultrafiltration Classification of CPT

The enzymatic hydrolysate supernatant 10% (*w*/*v*) was ultrafiltrated using the HYM-Multi-RN multifunctional laboratory separator (Ge, Pittsburgh, PA, USA). The membrane material was polysulfone. The supernatant was first processed through a 5000 NMWC cartridge (Model UFP-5-C-4X2MA, membrane area 1400 cm^2^) to separate the >5 kDa fraction (CPT1) from the <5 kDa permeate. The permeate was then further processed through a 3000 NMWC cartridge (Model UFP-3-C-4X2MA, membrane area 1400 cm^2^) to obtain the 3–5 kDa (CPT2) and <3 kDa (CPT3) fractions. After each run, the cartridges were cleaned by recirculating 0.5 mol/L NaOH for 1 h, followed by thorough rinsing with ultrapure water until the eluent pH returned to neutral. All fractions, along with the unfractionated hydrolysate, were lyophilized and stored for subsequent analysis.

### 2.4. Determination of Gln Content

The content of peptide-bound glutamine was determined by a difference method following selective conversion of glutamine residues to acid-stable 2,4-diaminobutyric acid (DABA) using bis-1,1-trifluoroacetoxy-iodobenz-ene (BTI) [15]. Briefly, 500 µL of sample (100 mg/mL) was added to an ampoule (Hawk, Harbin, China), mixed with 2 mL of 10 mg/mL BTI acetonitrile-aqueous solution, and incubated at 50 °C for 2 h. This step converts the amide side chain of glutamine to an amino group, yielding DABA, which does not generate glutamic acid during acid hydrolysis. An untreated sample served as the control. Both samples were then mixed with 5 mL of HCl (6 mol/L), and the ampoules were vacuum-sealed and hydrolyzed at 110 °C for 24 h. After cooling, pH was adjusted to 6–8 with NaOH, and glutamic acid content was measured using a glutamic acid biosensor analyzer. Glutamine content was calculated using Equation (1).

(1) $$Gln\;content\;(mg/mL)=C_{control}-C_{sample}$$

Here, C_control_ represents the glutamic acid content measured in the control group (sample hydrolyzed without BTI protection); C_sample_ represents the glutamic acid content measured in the sample group (sample hydrolyzed with BTI protection).

### 2.5. Methods for Structural Characterization of CPT Fractions

#### 2.5.1. Scanning Electron Microscopy (SEM)

The samples were sputter-coated with gold prior to observation and imaging using a field emission scanning electron microscope (S-4300 SEM, Hitachi High-Tech, Tokyo, Japan). The microscope was operated in high-vacuum mode at an accelerating voltage of 20.00 kV with magnifications set at 500× and 2000×.

#### 2.5.2. Fourier Transform Infrared Spectroscopy (FTIR)

The sample (1 mg) was thoroughly mixed with 200 mg of potassium bromide (KBr) and compressed into a transparent thin pellet. Subsequently, the Fourier transform infrared imaging system (Spotlight400, Perkin Elmer Inc., Shelton, CT, USA) was then used to acquire spectra over the range of 4000 to 500 cm^−1^. The secondary structures of each fraction were analyzed via PeakFit 4.12 software, with primary focus on the amide I band [17].

#### 2.5.3. UV-Visible Spectrum Scanning Method

The sample (10 mg) was dissolved in 10 mL of distilled water and filtered through a 0.45 µm microporous membrane. Using distilled water as the blank control, a UV-visible spectrophotometer (TU-1901, Beijing Purkinje General Instrument Co., Ltd., Beijing, China) was employed to perform a full-wavelength scan over the range of 200–500 nm. The absorption peak intensities of the sample around 220 nm and 280 nm were then observed [18].

#### 2.5.4. Determination of Particle Size, Polydispersity Index (PDI), and Zeta Potential

The particle size, polydispersity index (PDI), and zeta potential of the samples were determined following the method of Wang et al. with slight modifications [19]. Briefly, 5 mg aliquots of the samples were dispersed in 10 mL of 0.01 mol/L sodium phosphate buffer (pH 7.0). Subsequently, the particle size distribution, PDI, and zeta potential were measured using a laser particle size analyzer (Zetasizer Nano ZS90, Malvern Instruments Co. Ltd., Malvern, Worcestershire, UK) based on dynamic light scattering (DLS) and laser Doppler electrophoresis (LDE) principles.

### 2.6. Determination of Functional Properties of CPT Fractions

#### 2.6.1. Viscosity Measurement

Viscosity was determined with minor modifications to the method reported by Deng et al. [20]. Briefly, a 1% (*w*/*v*) sample solution was prepared using distilled water. Subsequently, the viscosity of each treated component solution was measured at 25 °C using a rotational viscometer (Model NDJ-79, Pingxuan Scientific Instruments Co., Ltd., Shanghai, China).

#### 2.6.2. Determination of Temperature Stability and Industrial Sterilization Stability

Temperature stability was determined by the method of Kato and Wang et al. with slight modifications [21]. Protein samples at 2 mg/mL in 50 mM Tris-HCl buffer (pH 7.0) were heated at 50–90 °C for 20 min. Turbidity was measured as the absorbance at 550 nm.

For industrial sterilization stability: 5 mg of sample was accurately weighed and dissolved in 5 mL of distilled water. After high-pressure sterilization at 121 °C for 20 min, ultraviolet absorption spectroscopy was performed.

#### 2.6.3. Determination of Foaming Properties

Foaming capacity (FC) and foaming stability (FS) were determined following the method described by Deng et al. with slight modifications [20]. Briefly, 50 mL of sample solution (30 mg/mL) was prepared, homogenized at 9870× *g* (10,000 rpm) for 2 min using a high-speed homogenizer, and immediately transferred to a volumetric cylinder. After 5 min, the volume of the aerated sample was recorded. Foaming capacity was calculated using Equations (2) and (3), respectively.

(2) $$\mathrm{FC}\;(\%)=[(V_{1}-V_{0})/V_{0}]\;\times \;100$$

(3) $$\mathrm{FS}\;(\%)=[(V_{2}-V_{0})/(V_{1}-V_{0})]\;\times \;100$$

Here, V_0_ represents the volume before whipping (mL), V_1_ is the volume measured after 2 min of homogenization, and V_2_ is the volume measured after the solution had stood still for 5 min after homogenization.

#### 2.6.4. Determination of Emulsification Properties

Emulsifying activity index (EAI) and emulsifying stability index (ESI) were determined following the method described by Cao et al. with slight modifications [22]. Briefly, 1.0 mL of corn oil and 3.0 mL of 1% (*w*/*v*) protein solution (dissolved in 0.1 mol/L phosphate buffer) were mixed by shaking and homogenized at 9870× *g* (10,000 rpm) for 1 min. At predetermined time intervals, a 50 µL aliquot of the emulsion was withdrawn from the bottom of the container and diluted with 5 mL of 0.1% (*w*/*v*) SDS. The absorbance of the diluted emulsion was measured at 500 nm. EAI and ESI were calculated using Equations (4) and (5), respectively.

(4) $$\mathrm{EAI}\;(m^{2}/g)=(2\times 2.303\times A_{0}\times D)/(C\times \varphi \times 10000)$$

(5) $$\mathrm{ESI}\;(\min)=(A_{0}\times 10)/(A_{0}-A_{10})$$

Here, D is the dilution ratio; A_0_ and A_10_ refer to the absorbance at 0 and 10 min, respectively; C represents the sample mass concentration (mg/mL); φ represents the optical path, φ = 0.01 m; and V represents the volume fraction of the oil phase, V = 0.25.

### 2.7. Determination of Antioxidant Properties of CPT Fractions

#### 2.7.1. Determination of ABTS Radical Scavenging Activity

ABTS radical scavenging activity was determined following the methods described by Chen et al. with minor modifications [23]. Briefly, an ABTS stock solution was prepared by mixing 7 mM of ABTS solution and 2.45 mM of potassium persulfate solution at a volume ratio of 2:1 (*v*/*v*), then incubated in the dark for 12–16 h. Prior to the assay, the stock solution was diluted with PBS (pH 7.4) to an absorbance of 0.700 ± 0.02 at 734 nm. Then, 100 µL of sample or distilled water (blank control) was mixed with 100 µL of the ABTS working solution, and incubated at room temperature for 6 min. Ascorbic acid was used as the positive control. The absorbance was measured at 734 nm, and the ABTS radical scavenging activity was calculated using Equation (6).

(6) $$\mathrm{ABTS}\;\mathrm{radical}\;\mathrm{scavenging}\;\mathrm{activity}\;(\%)=[(A_{0}-A_{1})/A_{0}]\;\times \;100$$

Here, A_0_ and A_1_ represent the absorbances of the blank control and the sample, respectively.

#### 2.7.2. Determination of Hydroxyl Radical Scavenging Activity

Hydroxyl radical scavenging activity was determined with slight modifications to the method reported by Li et al. [24]. Briefly, 1 mL of sample solution was mixed with 0.5 mL of 9 mmol/L FeSO_4_, 1 mL of 9 mmol/L salicylic acid solution (prepared in ethanol), 1 mL of 4.4 mmol/L H_2_O_2_, and 2 mL of distilled water. The mixture was incubated at 37 °C for 30 min, after which its absorbance was measured at 510 nm. Hydroxyl radical scavenging activity was calculated using Equation (7):

(7) $$\mathrm{Hydroxyl}\;\mathrm{radical}\;\mathrm{scavenging}\;\mathrm{activity}\;(\%)=[1-(A_{1}-A_{2})/A_{0}]\;\times \;100$$

Here, A_0_ is the absorbance of the blank solution, A_1_ is the absorbance of the sample, and A_2_ is the absorbance of the sample without H_2_O_2_.

### 2.8. Physicochemical and Structural Properties of the Identified Peptides

#### 2.8.1. Select the CPT3 Component to Simulate Gastrointestinal Digestion

Prepare a 50 mg/mL solution of the CPT3 and use it for subsequent digestion. For gastric digestion, the pH was adjusted to 2.0 using 1 mol/L of HCl and 1 mol/L of NaOH, and the solution was pre-incubated at 37 °C for 5 min. Pepsin (3000 U/mg; Macklin, Shanghai, China) was added at 3% (*w*/*w*), and the mixture was incubated at 37 °C for 3 h with gentle shaking, followed by boiling for 10 min to terminate the reaction. After cooling, one aliquot of the gastric digest was immediately subjected to intestinal digestion. For intestinal digestion, the pH of the gastric digest was adjusted to 6.8, pre-incubated at 37 °C for 5 min, and incubated with 3% (*w*/*w*) trypsin (250 U/mg; Macklin, Shanghai, China) at 37 °C for 3 h. The reaction was terminated by boiling for 10 min, and the final hydrolysis products are freeze-dried for storage.

#### 2.8.2. LC-MS/MS Identification of CPT3 Before and After Simulated Gastrointestinal Digestion

Bioteach Pack Co., Ltd. (Beijing, China) was commissioned to identify peptide sequences in the intercepted fraction using Nano LC-MS/MS. The sample (10 mg) was dissolved in 200 µL of water, and 100 µL was ultrafiltered (10 kDa, 13,000× *g*, 10 min). The filtrate was washed three times, reduced with DTT (10 mmol/L, 56 °C, 1 h), alkylated with IAM (20 mmol/L, dark, 40 min), quenched, desalted on C18 Stage-Tips, and dried at 45 °C. Peptide concentration was measured at 205 nm. Separation used an EASY-nLC with C18 columns (300 µm × 5 mm trap; 150 µm × 170 mm analytical, 1.9 µm) and a 66-min gradient (4–95% B; A: 0.1% FA, B: 80% ACN/0.1% FA) at 600 nL/min. DDA-MS operated at full-scan resolution 120,000 (IT 20 ms, 100–1500 *m*/*z*) and MS/MS resolution 15,000 (IT 22 ms, cycle time 2 s, NCE 30).

#### 2.8.3. The Physicochemical Properties of the Samples After LC-MS/MS Mass Spectrometry Identification

A total of 10,489 peptide sequences were identified from the samples before digestion, and 6218 peptide sequences were identified from the samples after digestion. By comparing the samples before and after digestion, 1747 identical sequences, namely the resistant-to-gastrointestinal-digestion sequences, were selected, and from these, 205 peptide sequences containing glutamine (Q) were further screened out. The physicochemical properties and structural characteristics of the selected peptide segments were analyzed using the Peptides package in R language 2.4.6 and Expasy-ProtParam 8.5.6 tools. The evaluated parameters included molecular weight, hydrophobicity, Boman index, net charge, hmoment (α-helix), hmoment (β-sheet), instability index, aliphatic index and isoelectric point to analyze the potential relationship between them and the regulation of intestinal barrier dysfunction.

### 2.9. Statistical Analysis

Each experiment was repeated in triplicate (*n* = 3). The results were represented as mean ± standard deviation (SD). Graphs were generated using Origin 2021 software. Statistical comparisons were performed using one-way analysis of variance and Duncan’s test. SPSS Statistics 21.0 was used to analyze the data. Significant differences were considered at *p* < 0.05. IC_50_ values were determined by Probit regression using SPSS 21.0 software. Goodness-of-fit for each estimate is reported as 95% CI.

## 3. Results and Discussion

### 3.1. Analysis of Gln Content in CPT Fractions

Corn protein is commonly used as a high-quality raw material for preparing glutamine peptides due to its rich in Gln. Since the bioactivity of glutamine peptides is closely related to their Gln content, the Gln levels in each fraction obtained after ultrafiltration of CPT were first determined. The Gln content of CPT fractions is shown in Table 1. As the molecular weight decreased, the Gln content increased significantly, rising from 12.53% in CPT1 to 16.73% in CPT3. This phenomenon can be attributed to synergistic enzymatic hydrolysis, which selectively generates small Gln-containing peptides and free Gln. Combined with the molecular sieving effect of ultrafiltration, these small molecules pass through the membrane pores and are enriched in the low-molecular-weight fractions. Their high water solubility facilitates this enrichment process and ensures their stable presence in the aqueous system. Our previous studies have shown that CPT has a protective effect on ulcerative colitis mice and can protect the intestinal barrier function of Caco-2 cells by reducing inflammation and regulating tight junction proteins [11,15]. The enriched Gln content in CPT3 observed in our study suggests that this component may serve as a promising candidate for bioactivity studies.

To further evaluate the potential of this fraction, we also determined the mass yield and protein recovery of each fraction after ultrafiltration. The results showed that, relative to the corn protein hydrolysate before ultrafiltration, the mass yields of the fractions after ultrafiltration were 20.48% (CPT1), 16.98% (CPT2), and 61.27% (CPT3), respectively, and the corresponding peptide recoveries were 21.05% (CPT1), 14.82% (CPT2), and 60.72% (CPT3), respectively. These values also clearly demonstrate that CPT3 is the predominant fraction and should be prioritized in subsequent studies.

### 3.2. Structural Characterization of CPT Fractions

#### 3.2.1. Microscopic Morphology

Scanning electron microscopy is a technique specifically tailored for the analysis of material surface morphology, enabling clear observation of peptide surface characteristics [25].

**Figure 1:**
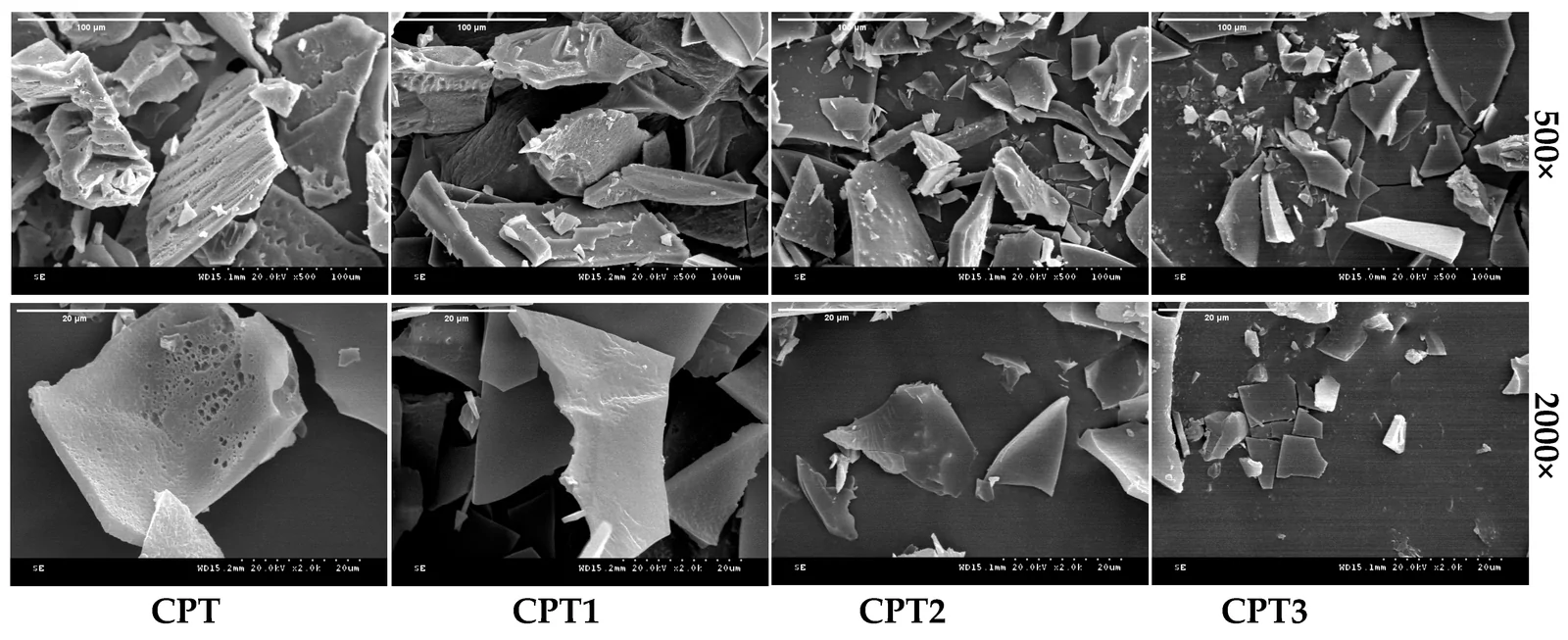
Scanning electron microscope images (SEM) images of CPT, CPT1, CPT2, and CPT3 at 500× and 2000× magnifications (scale bars = 100 µm and 20 µm, respectively).

As indicated in Figure 1, significant differences in surface morphology and porosity were observed among the samples. After enzymatic hydrolysis, CPT exhibited a loose, rough, and cracked structure, suggesting that proteases cleaved the peptide bonds. This cleavage caused the molecular chain to unfold and exposed hydrophobic amino acids, ultimately leading to the disintegration of the particles, resulting in a porous and rough structure. CPT1 exhibited a relatively thick sheet-like structure with a regular but slightly rough surface and exhibiting a high degree of aggregation. In contrast, CPT2 and CPT3 consisted of fine, dispersed fragmented structures with smaller particle sizes and increasingly dispersed distributions, due to the weak intermolecular forces of small peptides that inhibited aggregation. Similarly, previous research reported that Alcalase could effectively cleave pea, rice, and oat proteins into smaller molecular sizes [26].

In conclusion, the fractions obtained after enzymatic hydrolysis and ultrafiltration exhibit distinct morphologies, which in turn lead to differences in properties such as solubility. Since protein solubility is closely associated with other functional properties such as emulsifying activity, foaming capacity, and antioxidant activity, variations in microstructure are identified as a critical factor influencing the physicochemical and functional properties of the samples [27,28].

#### 3.2.2. Fourier Transform Infrared Spectral Analysis

Fourier Transform Infrared Spectroscopy (FTIR) is an important method for reflecting the secondary structure of proteins, which can provide information on the changes in relevant functional groups and secondary structures within proteins [29].

**Figure 2:**
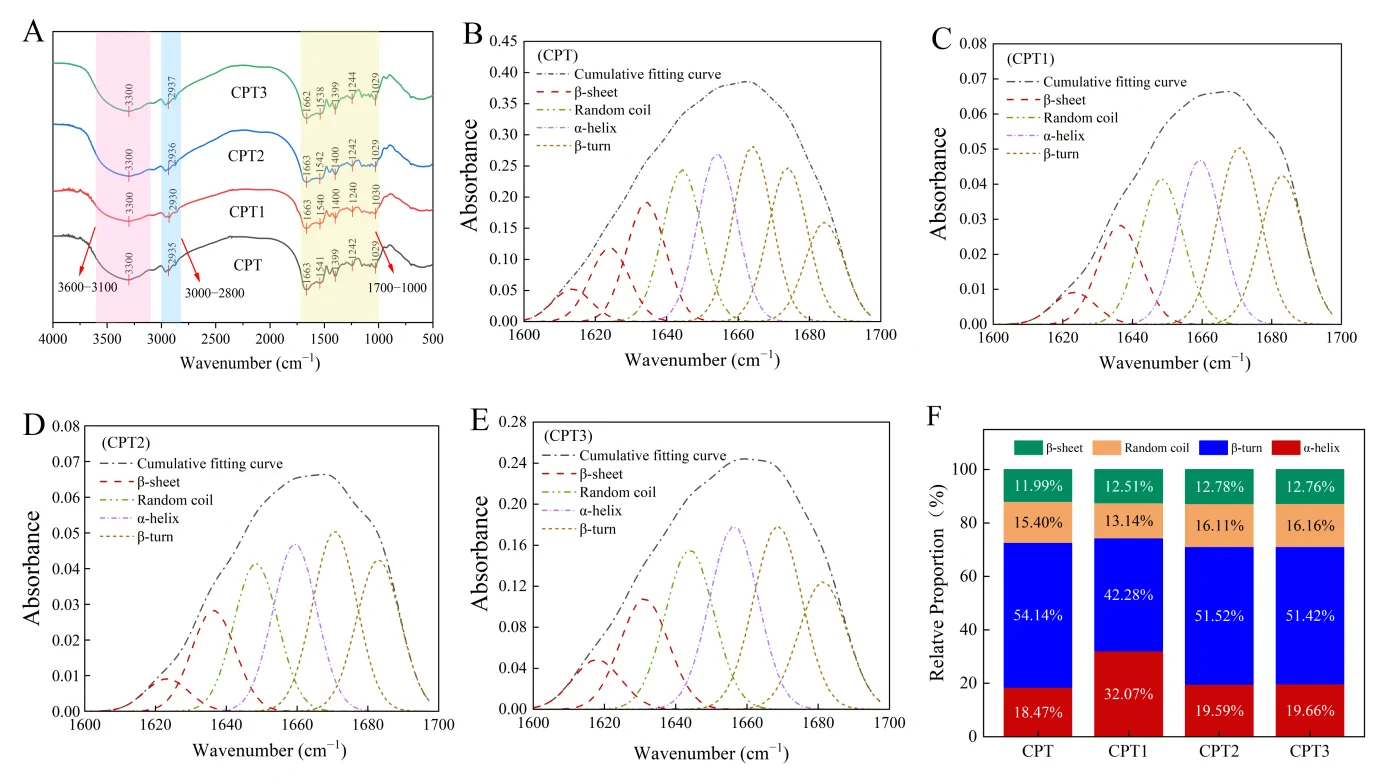
(**A**) Fourier transform infrared spectra (4000–500 cm^−1^). (**B**–**E**) Deconvolution of the amide I band (1600–1700 cm^−1^) for CPT (**B**), CPT1 (**C**), CPT2 (**D**), and CPT3 (**E**). (**F**) Relative proportions of secondary structure components (β-sheet, α-helix, β-turn, and random coil) derived from amide I band deconvolution.

As shown in Figure 2A, the range of 3500–3000 cm^−1^ was attributed to amide A, corresponding to O-H and N-H stretching vibrations related to hydrogen bonding. The broader amide A peak of CPT1 compared to CPT may be attributed to molecular aggregation induced by the removal of low-molecular-weight components during fractionation. This removal reduced the number of freely vibrating -OH and -NH_2_ groups on the surface, thereby altering the hydrogen bonding environment [30]. The 3000–2800 cm^−1^ range represented amide B, associated with C–H stretching in hydrophobic regions. Differences in peak position and shape among CPT, CPT1, CPT2, and CPT3 indicated distinct distributions of hydrophobic domains in hydrolysates with different molecular weights. CPT1 exhibited more pronounced hydrophobic characteristics, as evidenced by the distinct absorption features in the amide B region [31]. The ranges of 1600–1700 cm^−1^, 1500–1600 cm^−1^, and 1200–1400 cm^−1^ corresponded to amide I, II, and III, respectively [32]. All fractions showed clear absorption peaks, confirming the presence of amide bonds. Variations in peak position and intensity reflected differences in protein secondary structure. The overall absorption peak intensity of CPT2 and CPT3 was higher than that of CPT1 and CPT. Combined with the wavenumber distribution, it could be inferred that low-molecular-weight CPT2 and CPT3 contained a higher content of disordered secondary structures, whereas CPT1 had a higher proportion of ordered structures. In summary, FTIR spectral differences revealed variations in functional groups, hydrogen bonding, and secondary structure in hydrolysates of different molecular weights, providing a spectroscopic basis for further analysis. The remaining panels of Figure 2B–F, which present the deconvolution of the amide I band and the relative proportions of secondary structure components, are discussed in Section 3.2.3.

#### 3.2.3. Protein Secondary Structure

To quantitatively analyze the secondary structure composition, peak deconvolution of the amide I band was performed using PeakFit software (Figure 2B–E). This band contains ordered structures (α-helix, β-sheet) and disordered structures (β-turn, random coil). Ordered structures stabilize protein conformation via hydrogen bonding, whereas disordered structures are associated with the flexibility of hydrolysates [24]. The relative peak areas obtained from the deconvolution were used to estimate the proportions of each secondary structure type (Figure 2F).

As shown in Figure 2F, there are significant differences in the proportions of secondary structures among the components. The proportion of random coils increases as the molecular weight decreases, indicating that the structural disorder of the low-molecular-weight components is enhanced, which is consistent with the results of infrared spectral analysis. CPT is mainly composed of β-sheets (43.96%), with strong structural rigidity and high order. In contrast, CPT1 has the highest proportion of β-turns (44.11%) and the lowest proportion of β-sheets (13.97%), indicating significantly enhanced structural flexibility. Compared with CPT1, CPT2 and CPT3 have higher contents of random coils and β-sheets and lower contents of β-turns, showing flexible, loose and disordered secondary structural characteristics. It is speculated that the flexible and loose structures of CPT2 and CPT3 may contribute to better digestibility and potentially different bioaccessibility, making them suitable candidates for specific food applications [33]. Consequently, given that a looser protein molecular structure facilitates the exposure of embedded hydrophobic sites, CPT2 and CPT3 are expected to exhibit higher surface hydrophobicity compared to CPT1 [34].

#### 3.2.4. UV-Visible Spectrum Scanning

UV spectroscopy is a useful tool for monitoring conformational changes in proteins by detecting the microenvironment of aromatic amino acid residues [35]. Specifically, absorption peaks in the 260–300 nm range are associated with aromatic amino acids such as tryptophan, tyrosine, and phenylalanine, and changes in this region can reflect alterations in their exposure or local environment.

**Figure 3:**
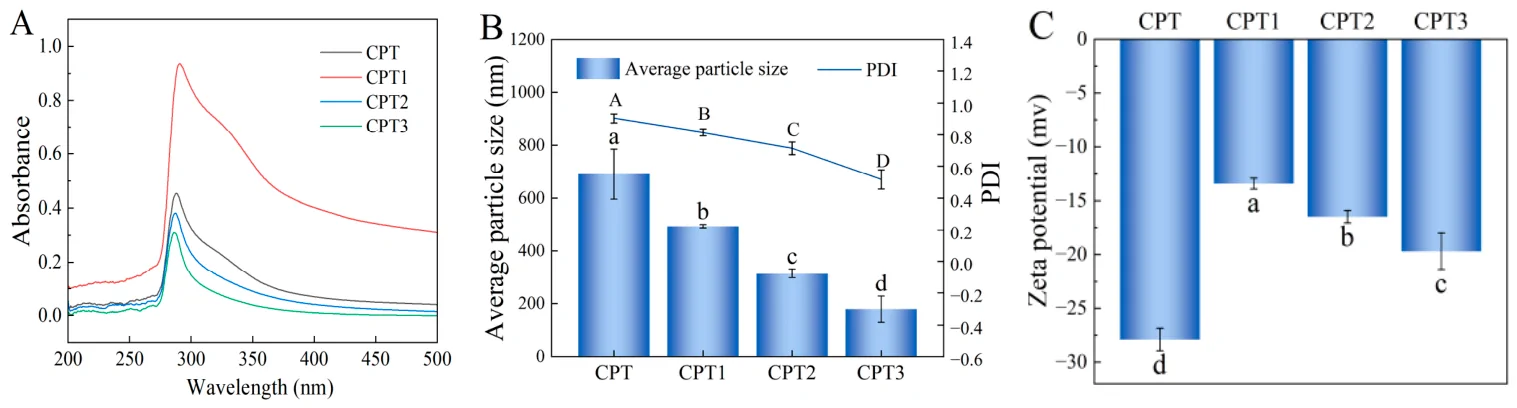
(**A**) UV-visible spectrum scanning. (**B**) Average particle size and polydispersity index (PDI). (**C**) Zeta potential. Data are expressed as mean ± SD (n = 3). Different letters indicate significant differences (*p* < 0.05).

As shown in Figure 3A, all CPT fractions exhibited similar absorption patterns in the 200–500 nm range, with characteristic peaks at 250–280 nm attributed to aromatic amino acids. CPT1 exhibited the highest absorption intensity, indicating a higher content or greater exposure of aromatic amino acid residues. In contrast, the absorbance of CPT, CPT2, and CPT3 decreased progressively, reflecting changes in the microenvironment or content of these residues induced by ultrafiltration fractionation. Furthermore, the absorbance of all samples decreased to nearly zero above 300 nm with no observable impurity peaks, confirming high sample purity and stable system conditions. These results are consistent with previous findings that enzymatic hydrolysis alters UV absorption profiles by modulating peptide conformation and the microenvironment of aromatic amino acid residues [24]. The remaining panels of Figure 3B,C, which present the average particle size, PDI, and zeta potential, are discussed in Section 3.2.5.

#### 3.2.5. Particle Size, Polydispersity Index (PDI), and Zeta Potential

Particle size and polydispersity index (PDI) are key indicators of the dispersion characteristics and uniformity of a substance, with lower PDI values indicating better dispersion uniformity [36]. Zeta potential, which characterizes electrostatic interactions on protein molecular surfaces, serves as an important parameter for evaluating colloidal stability and dispersibility [37,38].

As shown in Figure 3B,C, CPT3 showed the smallest particle size, lowest PDI, and highest zeta potential, indicating superior particle uniformity and colloidal stability. Ultrafiltration progressively reduced particle size by retaining larger peptide aggregates. This trend is consistent with previous studies on other plant-derived peptides, confirming the general applicability of ultrafiltration for regulating peptide particle size [39]. Furthermore, these results reveal an intrinsic correlation between the particle size of corn peptides and their molecular weight distribution and degree of aggregation. The reduced PDI values confirmed that fractionation improved the homogeneity of corn peptide dispersions. CPT3 has a higher Zeta potential, resulting in better dispersibility in water and less tendency for flocculation and sedimentation [40]. This is consistent with the findings of Ai et al., who reported that solutions with higher zeta potential values exhibited greater stability and smaller molecular sizes [41].

### 3.3. Functional Properties of CPT Fractions

#### 3.3.1. Viscosity

Understanding the viscosity characteristics of proteins is of great importance, as the viscosity properties of proteins significantly affect the flavor of foods, especially beverages [20].

**Figure 4:**
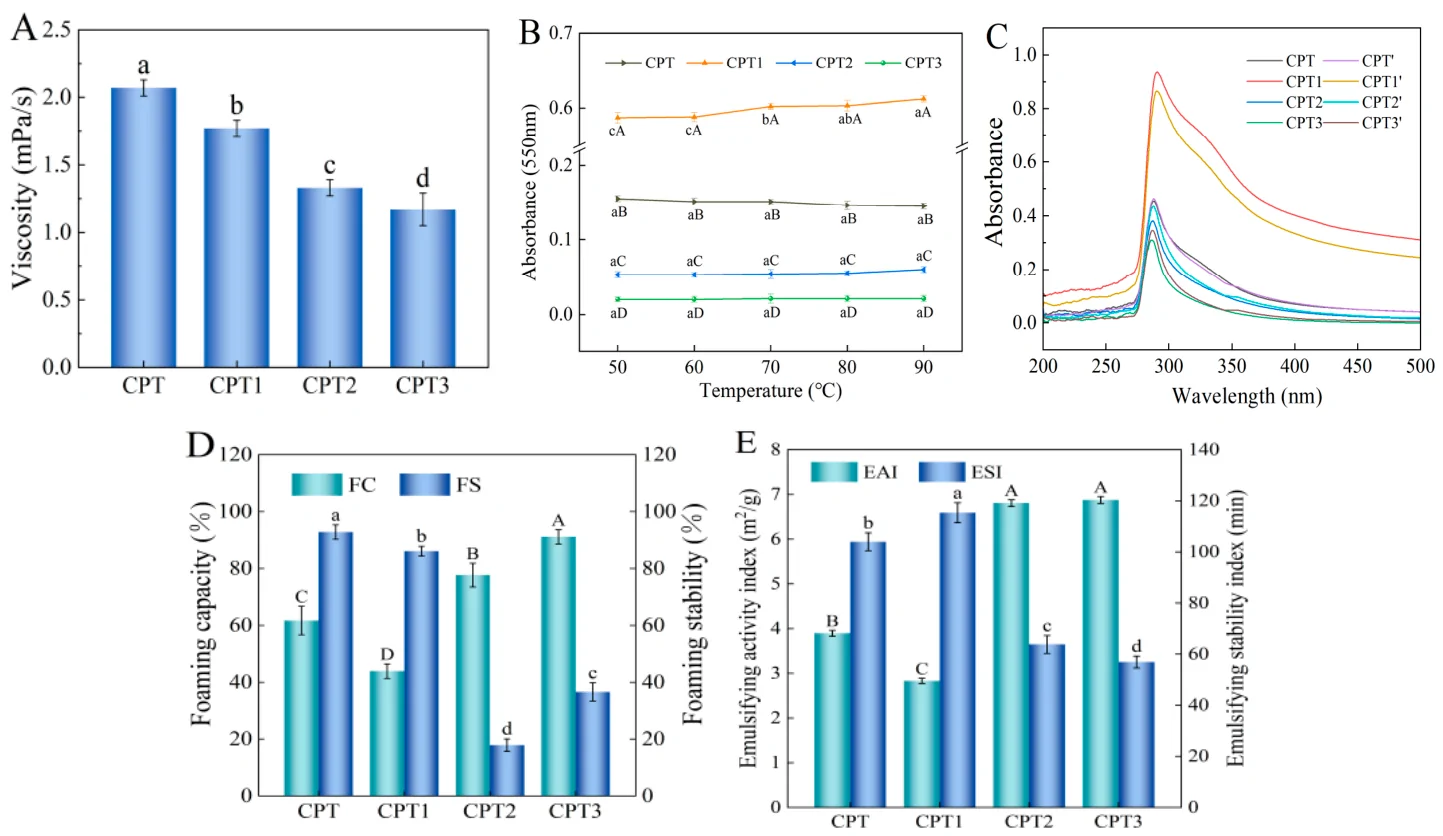
(**A**) Viscosity. (**B**) Temperature stability. (**C**) UV-visible absorption spectra of sample before and after industrial sterilization. (**D**) Foaming property. (**E**) Emulsifying property. Data are expressed as mean ± SD (n = 3). Different letters indicate significant differences (*p* < 0.05).

As shown in Figure 4A, the viscosity of fractions decreased with decreasing molecular weight. High-molecular-weight peptides exhibited higher viscosity due to longer chains, stronger intermolecular entanglement, and higher flow resistance. In contrast, low-molecular-weight peptide solutions exhibit low viscosity, primarily due to their short molecular chains, small spatial volume, weak intermolecular interactions, and consequently lower flow resistance [42]. This observation is consistent with Wu et al., who reported that the viscosity of high-molecular-weight proteins is proportional to their molecular weight [43]. The remaining panels of Figure 4 are discussed in the following sections: Figure 4B,C in Section 3.3.2, Figure 4D in Section 3.3.3, and Figure 4E in Section 3.3.4.

#### 3.3.2. Temperature Stability and Industrial Sterilization Stability

Given the prevalence of thermal processing in food manufacturing, the thermal stability of the samples was evaluated over a range of 50–90 °C, as well as under industrial sterilization conditions at 121 °C. Absorbance at 550 nm serves as an indicator of the degree of thermal aggregation of peptides, with lower absorbance corresponding to higher thermal stability [44]. Changes in spectral intensity and spectral shifts in the UV absorption spectrum can reflect structural changes and indicate the formation of new substances [45].

As shown in Figure 4B, CPT3 showed the lowest and most stable absorbance from 50 to 90 °C, while CPT1 exhibited the highest absorbance and poorest stability due to its larger molecular size and stronger aggregation tendency. CPT3 boasts advantages such as the smallest particle size, higher zeta potential, and optimal temperature stability. This finding is consistent with previous observations that modified proteins with smaller molecular sizes exhibit reduced aggregation and improved thermal stability, suggesting that the favorable temperature stability of the ultrafiltration fractions, particularly CPT3, is closely related to their low molecular weights. After sterilization at 121 °C for 20 min, the peak shape of the absorption spectrum of the samples did not change significantly, only the absorption intensity decreased (Figure 4C). This suggests that no major conformational changes occurred, and the impact on secondary structure was minimal, with the decreased absorbance likely resulting from slight degradation or concentration effects. Among the fractions, CPT3 exhibited the smallest decrease in absorption peak intensity, indicating the strongest stability under industrial sterilization conditions.

Overall, all components exhibited satisfactory thermal stability and industrial sterilization stability, with the low-molecular-weight and highly dispersed CPT3 demonstrating the most favorable performance.

#### 3.3.3. Foaming Properties

The dispersion phenomenon where bubbles form at the air–water interface is defined as foaming. As amphiphilic compounds, proteins adsorb rapidly at the interface to form viscoelastic films, which determines foaming performance [46].

As shown in Figure 4D, the foaming capacity of CPT fractions increased with decreasing molecular weight. Previous studies have demonstrated that the exposure of surface hydrophobic groups and enhanced solubility can promote the adsorption of proteins at the air–water interface, thereby improving foaming performance [47]. Therefore, CPT3 showed the strongest foaming capacity, which may be due to greater exposure of hydrophobic groups, lower surface tension, and faster interfacial adsorption. In contrast, CPT1, with a more ordered structure, exhibited lower foaming capacity due to fewer exposed hydrophobic groups. Similar results have been reported in relevant studies. For instance, highly hydrophobic oat peptides prepared from oat globulins exhibited strong foaming properties [33]. CPT and CPT1 displayed better foaming stability, which may be due to their higher viscosity. This finding is consistent with previous studies reporting that protein solutions with higher viscosity exhibited better foaming stability [48].

#### 3.3.4. Emulsification Properties

Emulsifying activity refers to the ability of proteins to adsorb at the oil–water interface and form stable emulsions, while emulsion stability reflects the capacity to maintain structural integrity over time [47].

As shown in Figure 4E, the emulsifying activity of CPT fractions increased with decreasing molecular weight, whereas emulsion stability showed the opposite trend. Studies have verified that the emulsifying properties of proteins are regulated by multiple factors, including molecular weight, molecular flexibility, and particle size [49]. In this study, the enhanced emulsifying ability of peptides may be due to their rapid adsorption at the oil–water interface, forming a dense interfacial film. In contrast, the higher molecular weights fraction exhibits lower emulsifying ability, which is consistent with the findings of Xu et al., who reported that high-molecular-weight yak hide gelatin exhibited inferior emulsifying properties [50].

Regarding emulsifying stability, previous researchers have established a correlation between emulsion stability and particle size, with emulsion stability decreasing as particle size diminishes [22]. CPT1 showed higher emulsion stability, which may be ascribed to its more ordered structure, larger particle size, and suitable surface hydrophobicity. CPT3 exhibited lower stability, possibly due to overexposed hydrophobic groups that weakened interfacial adsorption [51]. This weakened adsorption impedes the binding of CPT3 to lipids during emulsion formation, hindering the construction of a stable emulsion system and ultimately leading to reduced emulsion stability, which aligns with the results of Surh et al. [52]. Additionally, relevant studies have reported that freeze-dried peanut protein isolate with a more regular structure has higher emulsion stability than its spray-dried counterpart [53].

### 3.4. Antioxidant Activity of CPT Fractions

#### 3.4.1. ABTS Radical Scavenging Activity

The ABTS radical scavenging activity was determined to evaluate antioxidant capacity. Antioxidants act as hydrogen donors to reduce ABTS radicals, thereby decreasing absorbance at 734 nm. The magnitude of this absorbance reduction directly reflects the antioxidant capacity of the sample [54].

**Figure 5:**
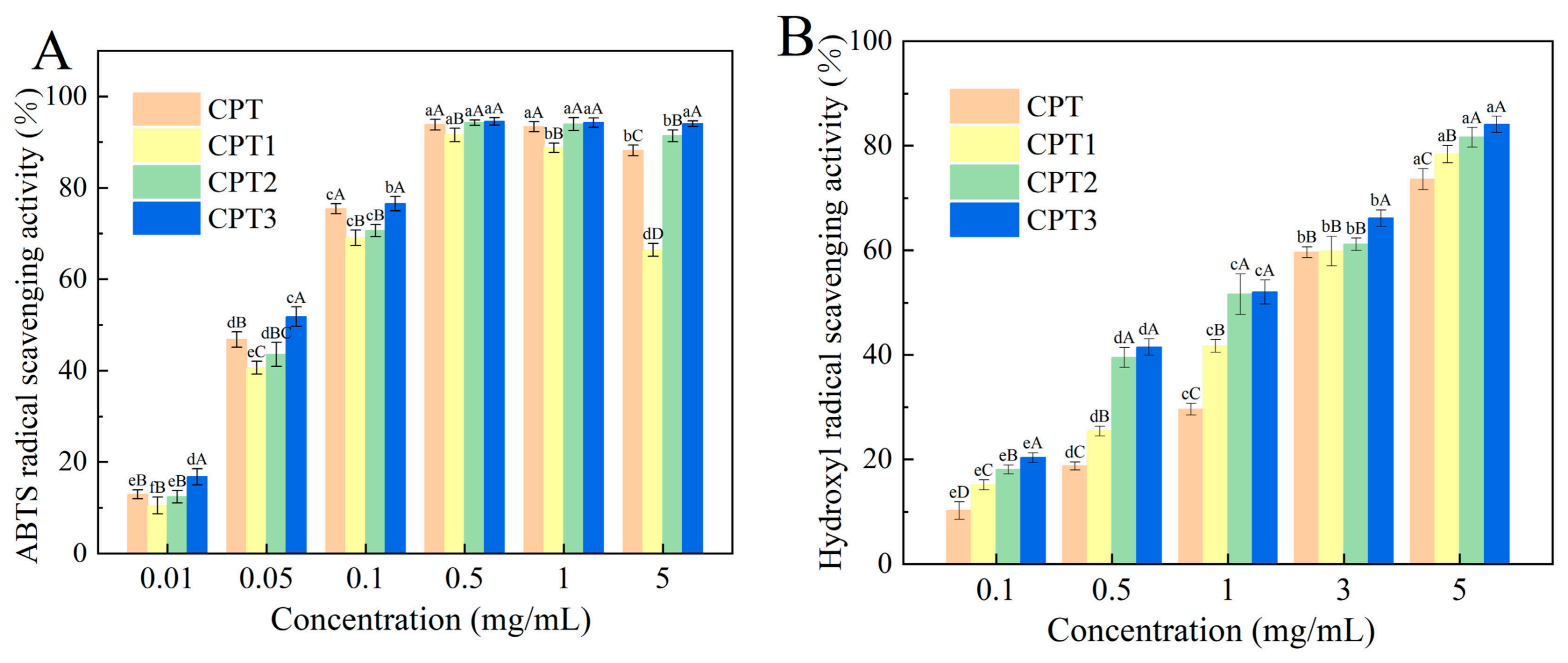
(**A**) ABTS radical scavenging activity. (**B**) Hydroxyl radical scavenging activity. Different lowercase letters (a–e) represent the differences among different concentrations for the same sample, while different uppercase letters (A–D) represent the differences among different samples at the same concentration. Data are expressed as mean ± SD (n = 3). Different letters indicate significant differences (*p* < 0.05).

As shown in Figure 5A, all fractions exhibited concentration-dependent ABTS scavenging activity. At the maximum concentration of 5 mg/mL, the scavenging rate of all components exceeded 80%. Based on IC_50_ values (Table 2), CPT3 showed the lowest value (0.042 ± 0.003 mg/mL), indicating the strongest activity. This value, while higher than that of ascorbic acid, still demonstrated high antioxidant potential. The superior scavenging ability of CPT3 may be attributed to the enhanced accessibility of small-molecular-weight peptides to water-soluble ABTS free radicals [55]. Moreover, the active functional groups of short peptide chains are more exposed, endowing them with more favorable antioxidant reaction kinetic characteristics [56]. This result is consistent with previous findings that small-molecular-weight peptides generally exhibit stronger antioxidant activity, and a negative correlation exists between peptide molecular weight and ABTS radical scavenging activity [57].

#### 3.4.2. Hydroxyl Radical Scavenging Activity

Hydroxyl radicals are highly reactive oxygen-free radicals that can cause oxidative damage to biological molecules and trigger chronic diseases [58]. Therefore, eliminating hydroxyl radicals is an important antioxidant mechanism.

As shown in Figure 5B, all fractions exhibited concentration-dependent hydroxyl radical scavenging activity from 0.1 to 5 mg/mL. CPT3 showed significantly higher activity than the other fractions at the same concentration. According to IC_50_ values (Table 2), ascorbic acid (0.065 ± 0.004 mg/mL) displayed the strongest activity, followed by CPT3 (0.807 ± 0.019 mg/mL), while CPT showed the lowest activity. This suggests that there are differences in the types or contents of active components among different fractions, which may be attributed to the different enrichment of active substances during the fractionation process. This indicates that CPT3 may be enriched with components possessing higher hydroxyl radical scavenging capacity, and that the structure or mechanism of action of its active substances is more conducive to binding with hydroxyl radicals. Girgih et al. reported that the hydroxyl radical scavenging activity of ultrafiltered fractions was higher than that of unfractionated samples, which is consistent with the findings of the present study [59].

### 3.5. Analysis of the Identified Peptides

#### 3.5.1. Analysis of Amino Acid Composition of the Identified Peptides

The physiological functions of active peptides are closely related to their structural characteristics, such as the length of the peptide chain, structure and amino acid composition [60]. This study analyzed the amino acid composition of the selected peptides to explore the potential relationship between these structural characteristics and the regulation of intestinal barrier dysfunction.

A total of 205 glutamine peptides were identified (Table S1). Two metrics were employed to characterize the amino acid profiles: the percentage of peptide sequences containing each amino acid and the percentage of each amino acid among the total residues. The results showed that the hydrophobic amino acids were distributed to varying degrees. Among them, Pro, Leu, and Ile exhibited the highest occurrence frequencies, at 62.44%, 54.63%, and 43.41%, respectively, followed by Phe (19.02%), Cys (16.59%), Val (15.61%), and Ala (14.63%). In terms of total residue composition, apart from the most abundant glutamine (23.49%), Pro, Leu, and Ile also contributed substantially, accounting for 18.69%, 12.49%, and 9.61% of the total residues, respectively (Table 3). Hydrophobic amino acids are one of the core composition features of the overall sequence. Yang et al. demonstrated that high hydrophobic amino acid peptides can improve ulcerative colitis in mice by repairing the intestinal barrier, regulating the microbiota and amino acid metabolism [61]. This confirmed that hydrophobic amino acids such as Leu, Pro, and Ile are the core structural basis for barrier repair. At the same time, Gln provides crucial nutritional support for intestinal barrier repair. As the main energy source for intestinal mucosal epithelial cells, it can alleviate mucosal atrophy under the state of barrier disorder, promote intestinal villus growth, and regulate the balance of intestinal microbiota, thereby strengthening the biological barrier function of the intestine [62]. Therefore, it is speculated that the identified peptide sequences have good potential in regulating the disorder of intestinal barrier function.

#### 3.5.2. Analysis of Physicochemical and Structural Properties of the Identified Peptides

The structural characteristics of 205 peptides were analyzed by the Peptides package in R language and Expasy-ProtParam tool, including nine indicators such as molecular weight, hydrophobicity, Boman index, net charge, Hmoment (α-helix), Hmoment (β-sheet), instability index, aliphatic index, and isoelectric point, to explore the potential relationship between these peptides and the regulation of intestinal barrier dysfunction.

**Figure 6:**
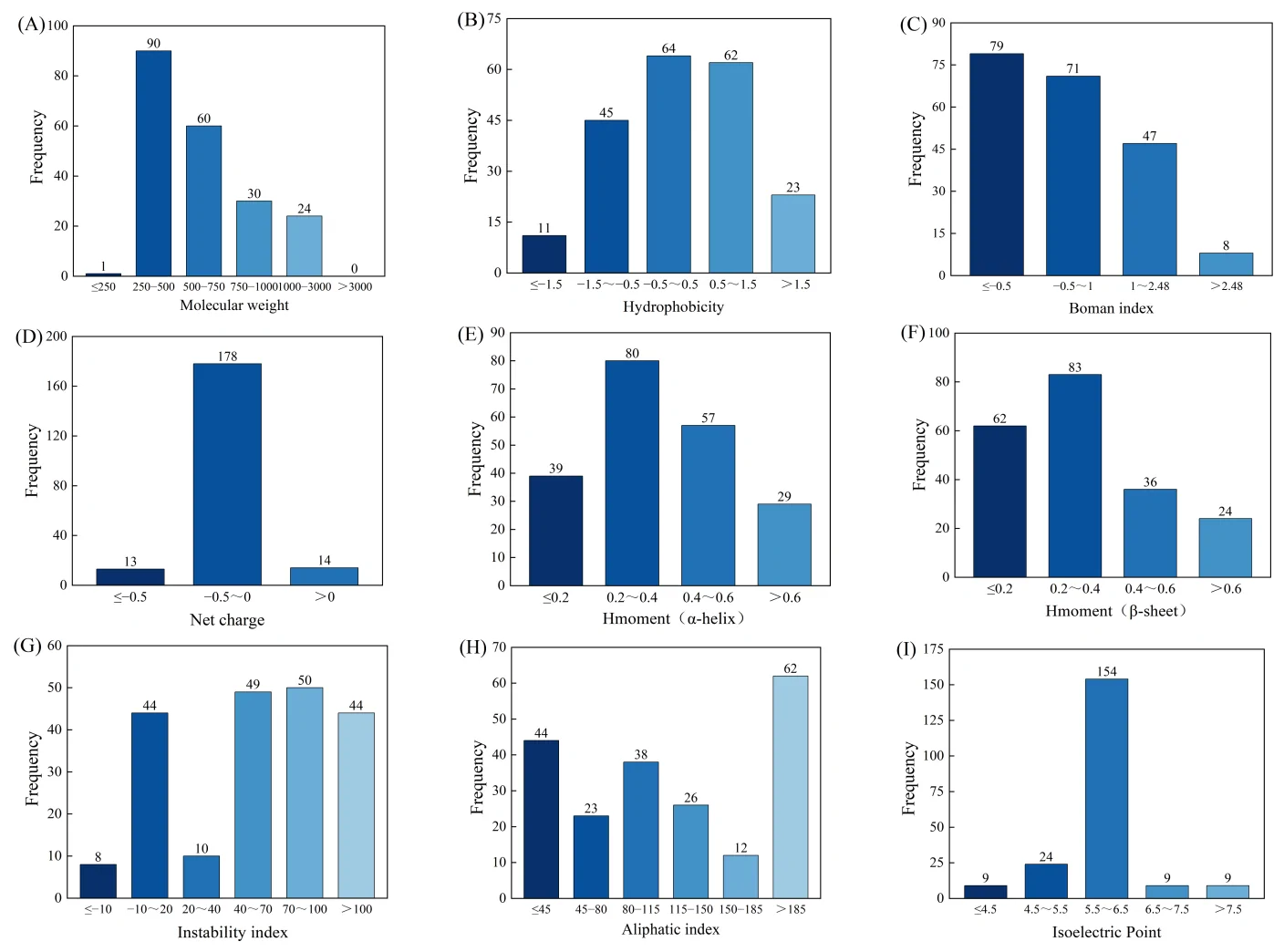
Analysis of structural characteristics of 205 corn glutamine peptides, including molecular weight (**A**), hydrophobicity (**B**), Boman index (**C**), net charge (**D**), Hmoment (α-helix) (**E**), Hmoment (β-sheet) (**F**), instability index (**G**), aliphatic index (**H**), and isoelectric point (**I**).

As shown in Figure 6A, the molecular weights of all the identified peptide molecules are all less than 3000 Da. Among them, the sequences of 181 peptides are less than 1000 Da, accounting for 88.29%. This is in line with previous results that low-molecular-weight peptides typically have higher bioavailability and membrane permeability. This molecular size limitation is particularly advantageous for intestinal absorption, as larger molecules encounter significant barriers when passing through the epithelial layer [63]. Hydrophobicity can be used to measure the hydrophilicity and hydrophobic balance of the peptides [64]. In this study, the hydrophobicity range of the 205 peptide sequences was from −2.55 to 2.42. Among them, 85 hydrophobic peptides accounted for 41.46% of the total analysis, while 56 hydrophilic peptides accounted for 27.32% of the total analysis. The hydrophobicity index of the amphiphilic peptides ranged from −0.5 to 0.5. Among the identified peptides, 64 were amphiphilic peptides, accounting for 31.21%. Therefore, the identified sequences overall showed a dominant feature of weak hydrophobicity and amphiphilicity. Most of the peptides were hydrophobic, while there was a certain proportion of hydrophilic and strongly hydrophobic peptide segments. Peptides with high hydrophobicity can form stable secondary structures such as α-helix and β-sheet in the aqueous phase, thereby providing better stability and biological activity for the peptides [65]. The hydrophilic and hydrophobic regions present in the amphiphilic peptide segments can ensure solubility in the intestinal cavity environment. At the same time, through hydrophobic interactions, they penetrate the mucus layer and bind to the intestinal epithelial cell membrane, thereby upregulating the expression of tight junction proteins and reducing intestinal permeability, exerting the protective effect of the intestinal barrier.

The Boman index is a parameter used to evaluate the protein-binding potential of peptides based on their amino acid sequences. Generally, a Boman index value greater than 2.48 indicates a higher binding propensity and potential multifunctionality, whereas a lower value suggests weaker interactions. In this study, a threshold of 2.48, as adopted from previous work, was used as the reference for favorable binding potential [66]. The higher the index, the higher the binding potential. When the Boman index is greater than 2.48, it indicates that the peptide has a high potential for protein binding. As can be seen from Figure 6C, the Boman index of most of the identified peptides is less than 2.48, and the Boman index values of 55 peptides are greater than 1. Only 8 peptides have Boman index values greater than 2.48, indicating that these peptides may have good protein binding potential. Among the analyzed peptide sequences, nearly 178 neutral charged peptides are predominant, which dominates the distribution. This distribution characteristic indicates that most of the peptides in the sample exhibit weak electrostatic polarity under physiological pH, which may help them maintain structural stability in the intestinal environment and reduce the probability of rapid degradation. These neutral charged peptides are more likely to have mild interactions with intestinal epithelial cells, thus potentially serving as ligands for regulating the dysfunction of the intestinal barrier. The hydrophobic moment can quantitatively describe the amphiphilicity of the peptide, reflecting the structural characteristics of the interaction with the membrane, it usually includes α-helix and β-sheet [67]. As shown in Figure 6E,F, the hydrophobic moments (α-helix) and (β-sheet) of the main peptides range from 0.2 to 0.4. Among them, the hydrophobic moments (α-helix) of 80 peptides are between 0.2 and 0.4, and the hydrophobic moments (β-sheet) of 83 peptides are also between 0.2 and 0.4. By comparing the same peptide sequences in these two groups, there are 29 overlapping peptide sequences, indicating that these peptides have a good potential to form amphiphilic α-helix and β-sheets.

Studies have shown that if the instability index of the peptide is less than 40, it is generally considered to be stable [68]. Among the identified peptide sequences, the instability coefficients of 62 peptides were less than 40, indicating that these peptide sequences were relatively stable which is essential for maintaining functional integrity in the harsh gastrointestinal environment [69]. The fatty acid index can reflect the thermal stability of the peptides. The higher the fatty acid index, the better the thermal stability of the peptide sequence, and peptides with good thermal stability have better tolerance in high-temperature environments and thus have better application prospects [70]. The fatty acid index values of 205 peptide sequences range from 0 to 292.5. The proportion of peptides with an index value exceeding 80 reaches 66.67%, indicating that most peptide sequences have good thermal stability. The isoelectric point is defined as the pH value at which the net charge of the peptide segment in the solution is zero. As shown in Figure 6I, the isoelectric points of most peptides are concentrated between 5.5 and 7.5, indicating that these peptides have good solubility under neutral conditions.

In summary, through bioinformatics analysis, the physical and chemical properties and structural characteristics of 205 identified peptides were determined. The study found that the molecular weights of all the identified peptides were mostly less than 1000 Da, and most of them had a negative net charge and a high Boman index and contained high hydrophobicity and amphiphilic peptides. This indicates that the 205 identified peptide sequences have good physical and chemical properties. Based on the above data, the 205 identified peptides can be regarded as potential ligands for regulating the dysfunction of the intestinal barrier.

## 4. Conclusions

This study systematically evaluated the physicochemical properties, functional characteristics, and in vitro antioxidant activities of corn protein hydrolysates and their ultrafiltration fractions. The results confirmed that ultrafiltration fractionation effectively modulates the functional and antioxidant properties of the hydrolysates. The shortening of peptide chains and the formation of a loose molecular structure are the core structural bases for the improvement of the functional properties and antioxidant activities of the hydrolysate fractions. CPT and CPT1 exhibited better performance in terms of foaming and emulsifying stability. Meanwhile, CPT2 and CPT3 demonstrated higher foaming and emulsifying properties, zeta potential, along with smaller particle size and lower viscosity. In terms of antioxidant activity, CPT3 exhibited the strongest ABTS radical scavenging ability and hydroxyl radical scavenging capacity. Among all fractions, CPT3 presented the optimal comprehensive functional properties and the highest in vitro antioxidant activity, indicating its potential to be developed as a natural antioxidant. Furthermore, the 205 peptide sequences identified from CPT3 in this study contain a high proportion of hydrophobic amino acids and exhibit favorable physicochemical properties, making them potential ligands for modulating intestinal barrier dysfunction. These findings enrich the database of corn-derived functional glutamine peptides, provide a large sample pool for screening superior peptides with both antioxidant and intestinal barrier-protective functions, and offer important theoretical and practical support for subsequent research on the mechanisms of peptide action, high-value utilization of corn proteins, and the development of intestinal-protective functional foods. Future studies may employ cellular or animal models to validate their intestinal barrier-protective effects and dose response relationships, further investigate the molecular mechanisms by which superior peptides regulate intestinal barrier function, and elucidate their interaction modes with key intestinal targets.

## Figures and Tables

**Table 1:** Gln content of CPT fractions.

Fraction	CPT	CPT1	CPT2	CPT3
Gln content (%)	12.53 ± 0.59 ^c^	15.28 ± 0.34 ^b^	14.76 ± 0.38 ^b^	16.73 ± 0.31 ^a^

Results are expressed as mean ± standard deviation (n = 3). Different lowercase letters indicate significant differences among treatments (*p* < 0.05).

**Table 2:** IC_50_ of ABTS radical and hydroxyl radical scavenging rate of CPT fractions.

Fraction	ABTS (mg/mL)	Hydroxyl Radical (mg/mL)
Ascorbic acid	0.002 ± 0.002 ^d^	0.065 ± 0.004 ^e^
CPT	0.050 ± 0.002 ^b^	2.000 ± 0.076 ^a^
CPT1	0.074 ± 0.007 ^a^	1.455 ± 0.109 ^b^
CPT2	0.057 ± 0.003 ^b^	1.120 ± 0.263 ^c^
CPT3	0.042 ± 0.003 ^c^	0.807 ± 0.019 ^d^

Results are expressed as mean ± standard deviation (n = 3); Different letters indicate significant differences among treatments (*p* < 0.05).

**Table 3:** Analysis of the amino acid composition of 205 peptides.

Amino Acid	Percentage of Peptides Containing Each Amino Acid (%)	Total Residue Frequency (%)	Amino Acid	Percentage of Peptides Containing Each Amino Acid (%)	Total Residue Frequency (%)
Ala (A) *	14.63	3.23	Gly (G)	23.41	5.24
Arg (R)	5.85	1.05	His (H)	5.85	1.48
Asn (N)	5.37	0.96	Ile (I) *	43.41	9.61
Asp (D)	2.93	0.52	Leu (L) *	54.63	12.49
Cys (C)	16.59	5.33	Lys (K)	0.98	0.17
Gln (Q)	100.00	23.49	Met (M) *	7.80	2.18
Glu (E)	4.88	0.96	Phe (F) *	19.02	3.41
Pro (P) *	62.44	18.69	Ser (S)	17.56	3.58
Thr (T)	9.27	1.75	Tyr (Y)	14.15	2.53
Val (V) *	15.61	2.97	Trp (W) *	1.95	0.35

Note: * represents hydrophobic amino acids. Hydrophobic amino acids were classified according to the Kyte–Doolittle hydrophobicity scale (positive GRAVY scores).

## Data Availability

The original contributions presented in this study are included in the article/Supplementary Material. Further inquiries can be directed to the corresponding author.

## Supplementary Materials

The following supporting information can be downloaded at https://www.mdpi.com/article/10.3390/foods15183283/s1, Table S1. Glutamine peptide sequence information identified in CPT3.
