# Supplementary material for: Molecular Weight-Dependent Functional and Antioxidant Properties of Glutamine-Rich Corn Protein Hydrolysate: Insights into the Predicted Structural Characteristics of Identified Peptides
Source: Foods. 2026 Sep 17;15(18):3283. doi: 10.3390/foods15183283 (PMC13606863; doi:10.3390/foods15183283)
Supplement: Supplementary file 1 [file foods-15-03283-s001.zip › foods-4548214-supplementary.pdf]

Table S1. Glutamine peptide sequence information identified in CPT3.

No.	Peptide sequence	calculated mass	m/z measured	score	intensity
1	PQPHPCPCQQPHPSPC	1922.7974	641.94104	56.48	1900000
2	PCPCQQPHPSPC	1463.5744	732.79547	51.9	492000
3	TQPPRPQPHPQPHPCPCQQPHPSPC	2961.3333	741.34052	51.72	894000
4	QQPHPSPCQLQGTC	1636.7086	819.36237	51.27	1200000
5	QYPTQPPQLSPCQQFGSC	2121.9248	1061.96912	51.17	15700000
6	QLQQPGPCPC	1183.5114	592.76355	47.66	22600000
7	QLQQPGPCPCNA	1368.5914	685.30334	46.38	2930000
8	QLQQPGPCPCN	1297.5543	649.7854	46.03	594000
9	RPQPHPQPHPCPCQQPHPSPC	2538.1216	635.53925	42.84	186000
10	QQPGPCPCNA	1127.4488	564.73267	41.15	1730000
11	CGLQQPTPCP	1156.5005	579.25806	40.85	31900000
12	LQQPGPCPCN	1169.4958	585.75543	40.77	808000
13	QQPGPCPCN	1056.4117	529.21387	40.52	311000
14	LSPCQQFGSC	1182.4798	592.2475	38.72	119000000
15	LQQPGPCPC	1055.4528	528.73425	38.29	30300000
16	SPCQQFGSC	1069.3957	535.70569	37.07	2800000
17	QQPTPCPY	989.4277	495.72131	36.36	1900000
18	PTQPPQLSPC	1123.5332	562.77435	36.26	26100000
19	QQPHPSPC	949.4076	475.71185	36.16	368000
20	MNPCMQYC	1102.3704	552.19312	35.44	3330000
21	LRQPQCSPL	1097.5652	549.7901	34.17	12000000
22	LSPCQQFGSCG	1239.5012	620.75848	33.36	591000
23	WQQPIVGGA	954.4923	955.50018	33.34	3820000
24	MQMPCPCAG	1066.3704	534.19281	32.73	4340000
25	QQPGPCPC	942.3688	472.19202	32.28	11200000
26	QYPTQPPQ	957.4556	479.7356	31.95	60800000
27	EEMQRPGTPL	1156.5546	579.28528	30.94	2510000
28	SDEIFQPK	962.4709	482.24274	30.56	320000
29	LSDEIFQPK	1075.5549	538.78497	30.51	519000
30	RQPQCSPLA	1055.5182	528.76715	29.95	2530000
31	QFLPPVTSM	1034.5107	518.26245	29.79	5810000
32	MQMPCPC	922.3169	462.16586	29.47	29500000

33	RQPQCSPL	984.4811	493.24881	28.96	76800000
34	FLTQPQ	732.3806	733.38873	27.92	1490000
35	RQQPIVGGA	924.5141	463.26471	27.43	129000
36	ENPALQPY	930.4447	466.22983	26.96	555000
37	SWQQPIVGGA	1041.5243	521.76941	26.58	1560000
38	YLPPQQQPQPW	1380.6826	691.34875	26.5	27000000
39	SLPHLTIQ	907.5127	454.76337	26.38	953000
40	LQ	259.1532	260.16055	26.19	295000
41	IQ	259.1532	260.16055	26.19	295000
42	QGL	316.1747	317.18213	25.49	121000
43	QGI	316.1747	317.18213	25.49	121000
44	YQQPIIGGA	945.492	473.74753	25.46	164000000
45	QMPCPC	791.2765	396.64563	25.28	4490000
46	LIQ	372.2373	373.24451	25.25	6150000
47	LLQ	372.2373	373.24451	25.25	6150000
48	ILQ	372.2373	373.24451	25.25	6150000
49	IIQ	372.2373	373.24451	25.25	6150000
50	IQA	330.1903	331.19769	25.14	327000
51	LQA	330.1903	331.19769	25.14	327000
52	LGQ	316.1747	317.18219	24.67	371000
53	IGQ	316.1747	317.18219	24.67	371000
54	SAAYPQ	635.2915	636.29962	24.6	2800000
55	QL	259.1532	260.16074	24.52	333000
56	PQ	243.1219	244.12938	24.42	53300000
57	FPQ	390.1903	391.19775	24.03	75900000
58	MMPQ	505.2029	506.21091	23.85	10500000
59	LLGQ	429.2587	430.26642	23.56	1240000
60	LIGQ	429.2587	430.26642	23.56	1240000
61	ILGQ	429.2587	430.26642	23.56	1240000
62	IIGQ	429.2587	430.26642	23.56	1240000
63	YPQ	406.1852	407.19305	23.25	33500000
64	MQIE	519.2363	520.24414	23.04	6060000
65	MQLE	519.2363	520.24414	23.04	6060000
66	QDL	374.1801	375.18759	22.96	200000
67	QDI	374.1801	375.18759	22.96	200000
68	MQRPGTPL	898.4695	450.24188	22.83	1270000
69	ILYQ	535.3006	536.30823	22.81	33200

70	LIYQ	535.3006	536.30823	22.81	33200
71	LLYQ	535.3006	536.30823	22.81	33200
72	IIYQ	535.3006	536.30823	22.81	33200
73	MNPALQQP	897.4378	449.7262	22.74	169000
74	VLQ	358.2216	359.2291	22.26	146000
75	VIQ	358.2216	359.2291	22.26	146000
76	LFPQ	503.2744	504.28195	22.25	730000000
77	IFPQ	503.2744	504.28195	22.25	730000000
78	QFLP	503.2744	504.2818	22.16	2030000000
79	QFIP	503.2744	504.2818	22.16	2030000000
80	TIFPQ	604.322	605.32953	21.86	6220000000
81	LFQP	503.2744	504.28174	21.65	15300000
82	IFQP	503.2744	504.28174	21.65	15300000
83	FIQ	406.2216	407.22906	21.62	3240000
84	FLQ	406.2216	407.22906	21.62	3240000
85	YLQ	422.2165	423.22458	21.56	86200
86	YIQ	422.2165	423.22458	21.56	86200
87	LSLQ	459.2693	460.27695	21.15	8180000
88	LSIQ	459.2693	460.27695	21.15	8180000
89	ISLQ	459.2693	460.27695	21.15	8180000
90	ISIQ	459.2693	460.27695	21.15	8180000
91	LLVQ	471.3057	472.31302	21.07	4980000
92	ILVQ	471.3057	472.31302	21.07	4980000
93	IIVQ	471.3057	472.31302	21.07	4980000
94	LIVQ	471.3057	472.31302	21.07	4980000
95	QLP	356.206	357.21335	20.91	40500000
96	QIP	356.206	357.21335	20.91	40500000
97	VAYPQ	576.2907	577.29865	20.75	12500000
98	QQPIIGGA	782.4286	392.22141	20.69	12600000
99	VVFPQ	588.3271	589.33441	20.64	4240000
100	IFQ	406.2216	407.229	20.54	491000
101	LFQ	406.2216	407.229	20.54	491000
102	LPQ	356.206	357.21347	20.42	51400000
103	IPQ	356.206	357.21347	20.42	51400000
104	PPQ	340.1747	341.18213	20.15	4340000
105	AYPQ	477.2223	478.23019	20.04	16800000
106	APSAIIPQ	795.449	398.73187	20.01	1380000

107	GQRAPPGPCPC	1195.5226	598.76331	19.96	1930000
108	LLIQ	485.3213	486.32889	19.9	2450000
109	LLLQ	485.3213	486.32889	19.9	2450000
110	LIIQ	485.3213	486.32889	19.9	2450000
111	ILLQ	485.3213	486.32889	19.9	2450000
112	IILQ	485.3213	486.32889	19.9	2450000
113	ILIQ	485.3213	486.32889	19.9	2450000
114	LILQ	485.3213	486.32889	19.9	2450000
115	FVQ	392.206	393.21332	19.87	921000
116	QLPSYRTNPCGV	1390.6663	696.341	19.74	1000000
117	QAIA	401.2274	402.23505	19.68	345000
118	QALA	401.2274	402.23505	19.68	345000
119	FAQ	364.1747	365.18225	19.56	32900
120	PVMMPQ	701.324	351.66919	19.42	3740000
121	QQPIVGGA	768.413	385.21371	19.36	2840000
122	HQIP	493.2649	494.2728	19.33	2430000
123	HQLP	493.2649	494.2728	19.33	2430000
124	RFEQGDVV	948.4665	475.24069	19.32	581000
125	QSW	419.1805	420.18811	19.2	4190000
126	VYPQ	505.2536	506.26141	19.19	71100
127	FVQPA	560.2958	561.30353	19.03	3040000
128	MPQ	374.1624	375.17007	18.91	570000
129	LSVQ	445.2536	446.26096	18.9	1470000
130	ISVQ	445.2536	446.26096	18.9	1470000
131	IVLQ	471.3057	472.31296	18.85	5030000
132	LVLQ	471.3057	472.31296	18.85	5030000
133	IVIQ	471.3057	472.31296	18.85	5030000
134	LVIQ	471.3057	472.31296	18.85	5030000
135	YQQPIIG	817.4334	818.44049	18.69	6210000
136	MMMPQ	636.2434	319.12866	18.5	327000
137	YQPL	519.2693	520.27692	18.44	64700000
138	YQPI	519.2693	520.27692	18.44	64700000
139	SVCENPILQP	1155.5594	578.78662	18.37	375000
140	FLLPQ	616.3584	617.36584	18.36	2990000000
141	FIIPQ	616.3584	617.36584	18.36	2990000000
142	LLPQ	469.29	470.2977	18.24	11700000
143	IIPQ	469.29	470.2977	18.24	11700000

144	LIPQ	469.29	470.2977	18.24	11700000
145	ILPQ	469.29	470.2977	18.24	11700000
146	SPLQ	443.238	444.24579	18.22	1920000
147	SPIQ	443.238	444.24579	18.22	1920000
148	QVLSP	542.3064	543.31409	18.21	31200000
149	FLEQ	535.2642	536.27191	18.12	78300
150	FIEQ	535.2642	536.27191	18.12	78300
151	QILP	469.29	470.29764	18.1	4240000000
152	QLLP	469.29	470.29764	18.1	4240000000
153	QIIP	469.29	470.29764	18.1	4240000000
154	QLIP	469.29	470.29764	18.1	4240000000
155	QFLPA	574.3115	575.31891	17.98	407000000
156	LTTFPQ	705.3697	706.37756	17.91	14200000
157	QMPM	505.2029	506.21045	17.81	1240000
158	TLGDPQRPL	995.54	498.77771	17.77	334000
159	LGLQ	429.2587	430.26642	17.46	1240000
160	LGIQ	429.2587	430.26642	17.46	1240000
161	IGLQ	429.2587	430.26642	17.46	1240000
162	IGIQ	429.2587	430.26642	17.46	1240000
163	YQQPIIGG	874.4548	438.23471	17.4	13800000
164	LTIPQ	570.3377	571.3457	17.33	78700000
165	LGGQ	373.1961	374.1998	17.06	132000
166	IGGQ	373.1961	374.1998	17.06	132000
167	QPIIGG	583.3329	584.34015	17.02	10100000
168	AAYPQ	548.2594	275.13705	16.88	463000
169	LLQPQ	597.3486	598.35626	16.87	45300000
170	LIQPQ	597.3486	598.35626	16.87	45300000
171	YIHQ	559.2754	280.64532	16.73	82200
172	YLHQ	559.2754	280.64532	16.73	82200
173	PTQPPQ	666.3337	334.17422	16.61	138000
174	QQIP	484.2645	485.27225	16.58	26500000
175	QQLP	484.2645	485.27225	16.58	26500000
176	QLQ	387.2118	388.2193	16.52	165000
177	QIQ	387.2118	388.2193	16.52	165000
178	ASPLQPC	771.3585	386.68637	16.45	686000
179	YQQP	534.2438	535.25153	16.41	9370000
180	ATIFPQ	675.3592	676.367	16.41	15600000

181	VMMPQ	604.2713	303.1427	16.4	3360000
182	LLLPQ	582.3741	583.38135	16.27	17900000
183	LLIPQ	582.3741	583.38135	16.27	15600000
184	LILPQ	582.3741	583.38135	16.27	17900000
185	ILLPQ	582.3741	583.38135	16.27	17900000
186	LQLP	469.29	470.2973	16.09	125000000
187	IQLP	469.29	470.2973	16.09	125000000
188	LQIP	469.29	470.2973	16.09	125000000
189	IQIP	469.29	470.2973	16.09	125000000
190	SVGSQ	476.2231	477.23404	15.98	278000
191	CSQAPIA	745.3429	373.6788	15.83	11300000
192	LPQCL	629.3207	630.32544	15.79	1420000
193	LFLQ	519.3057	520.31329	15.78	1270000
194	IFLQ	519.3057	520.31329	15.78	1270000
195	IFIQ	519.3057	520.31329	15.78	1270000
196	LFIQ	519.3057	520.31329	15.78	1270000
197	LIVPQ	568.3584	569.36578	15.52	19700000
198	LLVPQ	568.3584	569.36578	15.52	19700000
199	QYGAPLLQ	888.4705	445.24252	15.3	434000
200	LLPFNQ	730.4014	366.20767	15.23	346000
201	QHQFLP	768.3919	385.20309	15.21	17600000
202	YVQFPQ	780.3806	391.19775	15.15	8610
203	QAAP	385.1961	386.20731	15.07	346000
204	PILGQ	526.3115	527.31879	15	4070000
205	PLLGQ	526.3115	527.31879	15	4070000
